# Strengthening event-based surveillance (EBS): a case study from Afghanistan

**DOI:** 10.1186/s13031-024-00598-1

**Published:** 2024-04-30

**Authors:** Mohamed Mostafa Tahoun, Mohammad Nadir Sahak, Muzhgan Habibi, Mohamad Jamaluddin Ahadi, Bahara Rasoly, Sabrina Shivji, Ahmed Taha Aboushady, Pierre Nabeth, Mahmoud Sadek, Alaa Abouzeid

**Affiliations:** 1 World Health Organization Country Office, Kabul, Afghanistan; 2 High Institute of Public Health, Alexandria University, Alexandria, Egypt; 3 Eastern Mediterranean Region WHO Office, Cairo, Egypt; 4 Ministry of Public Health, Kabul, Afghanistan; 5 United States Centers for Disease Control and Prevention, Atlanta, USA; 6 Division of infectious diseases, Brigham and women’s hospital, Harvard medical school, Boston, MA USA; 7 Faculty of Medicine, Cairo University, Cairo, Egypt

**Keywords:** Event-based surveillance, Early warning and response, Public health surveillance, And COVID-19

## Abstract

The sustained instability in Afghanistan, along with ongoing disease outbreaks and the impact of the COVID-19 pandemic, has significantly affected the country.

During the COVID-19 pandemic, the country’s detection and response capacities faced challenges. Case identification was done in all health facilities from primary to tertiary levels but neglected cases at the community level, resulting in undetected and uncontrolled transmission from communities. This emphasizes a missed opportunity for early detection that Event-Based Surveillance (EBS) could have facilitated.

Therefore, Afghanistan planned to strengthen the EBS component of the national public health surveillance system to enhance the capacity for the rapid detection and response to infectious disease outbreaks, including COVID-19 and other emerging diseases. This effort was undertaken to promptly mitigate the impact of such outbreaks.

We conducted a landscape assessment of Afghanistan’s public health surveillance system to identify the best way to enhance EBS, and then we crafted an implementation work plan. The work plan included the following steps: establishing an EBS multisectoral coordination and working group, identifying EBS information sources, prioritizing public health events of importance, defining signals, establishing reporting mechanisms, and developing standard operating procedures and training guides.

EBS is currently being piloted in seven provinces in Afghanistan. The lessons learned from the pilot phase will support its overall expansion throughout the country.

**Supplementary Information:**

The online version contains supplementary material available at 10.1186/s13031-024-00598-1.

## Background information

Following years of war and recent political changes, Afghanistan is facing a severe humanitarian crisis, with 24.4 million people needing humanitarian assistance and 18.1 million requiring health assistance to survive as of 2022. These challenges have heightened the risk of infectious disease outbreaks due to interrupted access to health services, increased food insecurity, internal displacement, and higher-than-usual rates of cross-border return (1). The country has recently experienced multiple outbreaks, including measles, diarrheal disease (including cholera), dengue fever, Crimean Congo hemorrhagic fever, pertussis, and malaria [1].

From the first reported case of COVID-19 in February 2020 to 3 December 2022, a total of 206,675 confirmed cases of COVID-19 and 7,840 deaths were reported [2]. However, the national serosurvey of COVID-19 morbidity in Afghanistan, which was conducted during June and July 2021, revealed that around 10 million people (31.5% of the population) were seropositive for antibodies against SARS-CoV-2, reflecting either current or previous COVID-19 infection [3].

Amidst Afghanistan’s complex challenges, the “One Health” concept stands out as essential, emphasizing the interdependence of human, animal, and environmental health. This approach promotes collaborative surveillance, integrating public health, veterinary, and environmental sciences to improve infectious disease detection, response, and management. Given Afghanistan’s vulnerability to health crises, adopting One Health and enhancing collaborative efforts are crucial for addressing immediate issues and building long-term resilience against future emergencies, ensuring a unified and effective approach to safeguarding health across all fronts.

The World Health Organization (WHO) recommends adopting an early warning and response (EWAR) function to enhance the capabilities of the surveillance system in detecting infectious diseases and outbreaks [4]. Event-based surveillance (EBS) is a crucial component of EWAR [5]. EBS helps rapidly detect unusual occurrences and outbreaks with pandemic potential, especially in areas with limited access to formal healthcare with a focus on all hazards approach, ensuring alignment with One Health concept. This rapid detection of potential events can trigger a quicker response than what might be expected from routine surveillance. For example, during a pandemic, EBS can detect new clusters and trigger response actions. EBS should work alongside case-based and laboratory surveillance systems such that individual cases identified in a cluster are referred to a health facility and have a sample collected, where appropriate. This link to case-based surveillance is essential to accurately count cases, track necessary epidemiologic and virologic information, and guide affected persons.

In Afghanistan, the National Disease Surveillance and Response (NDSR) system, operating under the General Directorate of Monitoring, Evaluation, and Health Information System, conducts infectious disease surveillance. This system includes indicator-based surveillance (IBS) and EBS components. The IBS component of NDSR gathers data on 17 notifiable diseases, prioritizing them based on preventability and public health importance from selected sentinel sites. However, EBS has been limited in scope and functionality, relying on few health facilities and communities through unstructured reporting.

During the onset of the COVID-19 pandemic, outbreak detection was primarily restricted to some health facilities later to all of them. The significance of EBS in Afghanistan became apparent following the escalation of the first COVID-19 wave in May 2020. During this period, numerous clusters of COVID-19 cases and deaths emerged within the community, yet they remained unreported and unmanaged. Consequently, the Ministry of Public Health (MoPH) surveillance department took action to enhance the EBS system.

The prioritization of EBS strengthening in Afghanistan, particularly in response to the COVID-19 pandemic, stems from its ability to capture a broader spectrum of signals related to disease activity, including outbreaks that might be missed or underreported in a weakened healthcare infrastructure. This approach compensates for the gaps left by traditional IBS, especially in scenarios where logistical, financial, or infrastructural constraints might hamper laboratory or case-based surveillance. By prioritizing EBS, Afghanistan aims to leverage its agility and broader scope to better navigate the complexities of disease surveillance in a context marked by significant disruptions to healthcare services and reporting mechanisms, thus offering a pragmatic path forward in bolstering the country’s disease outbreak response capabilities.

This paper highlights the EBS implementation process and shares lessons from an emergency country context. It presents the main steps and outputs of the first phase of EBS implementation in Afghanistan and discusses the subsequent steps in the implementation process.

## Methods

The implementation of the EBS system in Afghanistan started through collaboration between the MoPH, the WHO Country Office Afghanistan (WCO), the WHO Regional Office for the Eastern Mediterranean (EMRO), the United States Centers for Disease Control and Prevention (CDC), and the Global Health Development/Eastern Mediterranean Public Health Network (GHD|EMPHNET). The MoPH led the EBS enhancement process and implementation in the country with the technical support of WHO, the CDC, and GHD|EMPHNET.

The initial phase involved conducting a landscape assessment to better understand the existing surveillance system in Afghanistan, facilitating the design of an EBS that would integrate seamlessly. The landscape assessment was conducted through guided interviews with key surveillance officials at the MoPH and WCO using a questionnaire developed by the CDC. The questionnaire focused on gathering information related to detection, reporting, verification, and risk assessment of potential public health threats.

The results of the assessment were utilised in the second phase, involving the crafting of the EBS work plan. This work plan was drafted at country level with technical support from EMRO and CDC. The work plan outelined the implementation steps and activities aimed to enhance EBS in Afghanistan. These activities included identifying and training EBS mentors at the MoPH, mapping EBS stakeholders, identifying EBS information sources, prioritizing EBS events and defining signals, developing EBS guidelines and Standard Operating Procedures (SoPs), contextualizing EBS training materials, deploying the Epidemic Intelligence from Open Sources (EIOS) tool to implement the media scanning component of EBS, identifying EBS pilot sites (provinces), evaluating the pilot sites and expanding EBS throughout the country.

The MoPH surveillance department designated three mentors to enhance the EBS system. These mentors identified all relevant stakeholders for EBS in Afghanistan, referring to the existing IHR stakeholders list, and made necessary additions or exclusions to ensure representation for all an hazards approach through a multi-sectoral collaboration.

They assigned the mapped stakeholders to a coordination committee (CC). In the first meeting, the EBS mentors introduced EBS, explained its importance, and presented the EBS workplan to the CC. In a second meeting, the CC, guided by the EBS mentors, identified priority information sources for EBS. Subsequently, the CC established a smaller Technical Working Group (TWG) to prioritize events, develop signals, and create Standard Operating Procedures (SoPs) and training materials.

The TWG convened regular biweekly meetings with an initial focus on identifying priority events under EBS. Events are considered to be manifestations of disease or occurrences which have potential to create disease of known or unknown origin. “Event” in the event-based surveillance context is a signal that is verified to be truly occurring [4].

To facilitate the practice of prioritizing events, EBS mentors developed a complete list of events ahead of the meetings based on a risk assessment process using locally developed tool by WHO country office and MOH. No sensitivity analyses was done for any unusual event. The EBS mentors discussed the list with the TWG, and based on several criteria, including the potential impact of the event on public health, epidemic-prone diseases, historically prevalent diseases with the potential to re-emerge, and diseases targeted for eradication or elimination, priority events were selected.

After prioritizing events, the TWG created corresponding signals. Signlas are bits of information or occurences that may represent an acute risk to human health [4]. The TWG finalized the drafted signals with technical input from the WHO EMRO and US CDC. Following the creation of signals, the flow of information for EBS, focal points at each level, and roles and responsibilities were discussed by the TWG. The embedding of the EBS flow of information into the existing surveillance structure took place. Subsequently, all pieces developed for EBS were merged into a technical guideline with technical support from WHO, CDC, and GHD|EMPHNET. The selection of pilot sites for EBS implementation was carried out by the EBS CC.

## Key processes

### Landscape assessment

**Fig. 1:**
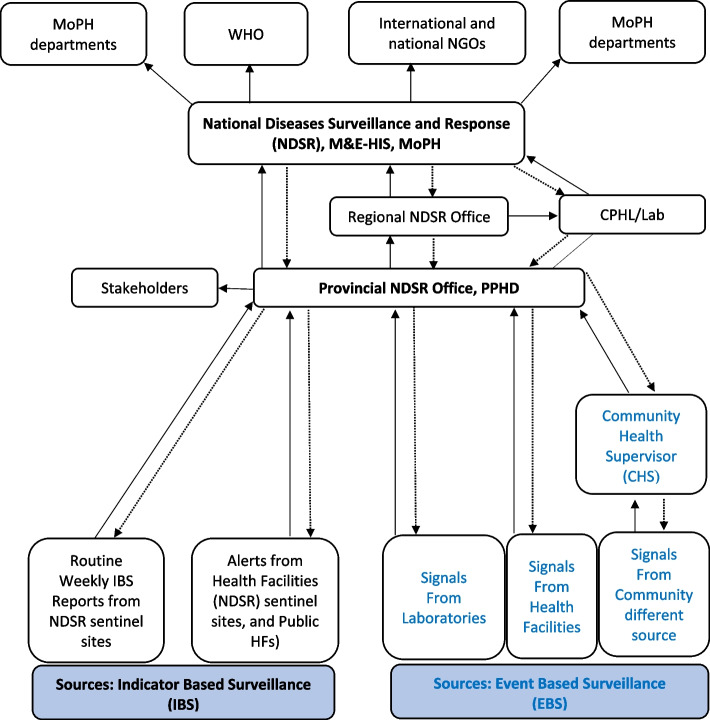
NDSR Structure and communication pathways in Afghanistan. NDSR Structure relies on IBS and EBS. IBS collects data from sentinel sites on priority disease. EBS (in blue) recentlcy been incorporated into NDSR structure. At the regional level and national level, NDSR offices disseminate information collected from EBS and IBS to relevant stakeholders, NGO’s, WHO and key department of Ministry of health

The landscape assessment of the surveillance system in Afghanistan highlighted the existence of an established National Disease Surveillance and Response (NDSR) system. NDSR includes different administrative levels (Fig. 1).

The NDSR functions at the national level and is responsible for the overall coordination of infectious diseases in the country. There are eight regional NDSR offices, each including several provinces, and their main responsibility is to coordinate the surveillance activities in their regions and report to the national surveillance office. Each province has its own surveillance office that is responsible for the implementation of surveillance activities in its province. In 2021, there were 519 sentinel sites (health facilities) (of 3750 health facilities countrywide) reporting to the NDSR system, including regional hospitals, provincial hospitals, district hospitals, and comprehensive and basic health facilities.

After the surge of the COVID-19 pandemic in May 2020, the country mandated both laboratory-confirmed and suspected clinical COVID-19 cases reporting from all health facilities daily using online reporting forms through a data capture tool developed using DHIS2.

The assessment found a well-established sentinel IBS within the NDSR. However, the EBS component was limited, relying on a small number of health facilities and communities as information sources without clear reporting mechanisms. Common signals such as hemorrhagic fever (1 and above), clusters of diarrhea (5+), and clusters of rash with fever (5+) in children were reported from these sources to provincial and national NDSR. The national NDSR then conducts outbreak investigations and laboratory confirmations.

Afghanistan’s challenging terrain, characterized by mountains, poses a unique challenge, with over 9 million individuals residing in under-served or hard-to-reach areas (defined as more than a one-hour walking distance to the nearest health facility). The IBS system gathers data solely from sentinel sites, not encompassing all health facilities, potentially leading to delays in case presentation at the nearest health facility due to both geographical barriers and the population’s health-seeking behavior.

Information in an EBS system can include informal sources such as media reports, reputable and well-connected community members, or other non-traditional sources for health information in addition to official or formal sources [4]. There was no specific and clear Community Event-Based Surveillance (CEBS), Health Facility Event-Based Surveillance (HEBS), Internet Event-Based Surveillance, laboratory, or veterinary EBS in place. Coordination with the animal health sector was limited to quarterly zoonotic committee meetings at the national and provincial levels and information sharing occured one way where only the MoPH surveillance department shared weekly surveillance information to animal health and other relevant sectors. There was no established reporting mechanism for disease and events from the private sector in the country. In addition, the lack of standardized guidelines and SoPs for EBS in Afghanistan posed a challenge for implementation and sustainability.

### Assigning national EBS mentors

CDC and WHO EMRO trained the three assigned mentors for EBS from the surveillance department of the MoPH on the principles and methods of EBS. The EBS mentors played an important role in advocating for and leading the integration of EBS in-country, coordinating with all stakeholders, and developing EBS guidelines and SoPs. In the second phase of EBS implementation, the mentors trained provincial and regional surveillance officers on EBS. EBS mentors intends to ensure the sustainability of event-based surveillance as part of the national disease surveillance strategy.

### Mapping the EBS stakeholders

Major stakeholders identified include the MoPH, Central Public Health Laboratory (CPHL), Ministry of Agriculture, Irrigation and Livestock (MAIL), Ministry of Borders and Tribal Affairs, Ministry of Hajj and Religious Affairs, Afghanistan Nuclear Energy Agency (ANEA), National Disaster Management Authority, National Environment Protection Agency, Civil Aviation Authority Afghanistan, International Organization for Migration (IOM), and the WHO Country Office in Afghanistan. Maintaining constant communications among these stakeholders involved establishing an EBS coordination committee (CC), led by the MoPH.

The CC, under the leadership of the MoPH, played a central role in mobilizing all necessary resources for EBS, identifying EBS information sources, and validating signals, guidelines, SoPs, training materials, data collection tools, and the work plan for EBS implementation. Additionally, the CC led the monitoring and evaluation of EBS implementation in the country.

To streamline focus on EBS activities, the TWG was established, comprising key focal points from the MoPH, MAIL, CPHL, and WCO. The TWG, through its collaboration, prioritized events, defined signals, developed EBS SoPs, contextualized training materials, and identified sites for EBS implementation.

### The information sources for EBS

EBS requires multi-sectoral collaboration and coordination between different stakeholders and relies on information sources beyond the traditional health system. While these may be directly linked to human health, data can also be provided by the non-human health sector, local communities, media, and international sources. Some information sources have key informants, who generally are community members with strong ties within their communities, such as religious leaders, schoolteachers, or even shop owners. The EBS CC identified and prioritized the EBS information sources in Afghanistan as described in Table 1 below.

**Table 1:** Information sources for EBS in Afghanistan

Information Source	Description and key informants
Community	Community-based surveillance is the systematic detection and reporting of events of public health significance within a community, by community members. Community health supervisors (CHSs) and community health workers (CHWs), religious leaders (Mullas) and school teachers are engaged and trained to detect and immediately report EBS signals or potential public health risks occurring in their communities.
Health Facility	Healthcare professionals are involved as primary reporting sources, recognizing signals during patient consultations, or as secondary sources reporting unusual occurrences related to human health or health risks picked up through patient consultations.
Animal health	Animal health surveillance is parallel to the human health system and collects information on the signals of animal disease that may be important to humans. This can occur through farmers, animal health workers in the community, wildlife health laboratories, and veterinary clinics, among others.
Laboratories	Laboratories, through the laboratory technicians, contribute to EBS through the detection and reporting of pathogens that are immediately notifiable, unusual patterns, unexpected increases in the detection of specific organisms, novel pathogens, and other signals.
Monitoring media through EIOS	Monitoring media can be a useful supplement to the community, health facility, and intermediate level components of the EBS system and can be useful to monitor the course of an event.
Point of Entry (PoE)	To prevent the cross-border spread of disease, PoE authorities will be among information sources for EBS, and the POE surveillance will be integrated with the overall surveillance system.

### Identify priority events and define signals

The prioritized events for EBS in Afghanistan include clusters of COVID-19 cases, avian influenza, acute hemorrhagic fever, vaccine-preventable diseases such as measles and poliomyelitis, cholera, norovirus (food poisoning), zoonotic priority diseases causing outbreaks, and emerging new diseases. The EBS TWG defined signals for each of these prioritized events and identified information sources, with key members from the Ministry of Education, municipality, Haj and Awkaf, Community Based Health Care department of the MoPH, MAIL, and other relevant stakeholders. These signals are standardized across the country.

Table 2 displays the list of signals defined for communities, health facilities, laboratories, and veterinary services.

**Table 2:** List of signals in Afghanistan

Community
1. 5 or more than five cases of fever or one death within a week from the same community
2. 5 or more than five cases of fever and cough or one death within a week from the same community
3. Two or more than two cases of fever with the skin rash or one death within a week from the same community
4. One or more cases of fever with unexplained purple or black and blue spots on the skin (Echymosis or petechial rash) or one death
5. Any child less than 15 years old having sudden weakness of limb/limbs
6. Two or more than two cases of fever with yellow eye or one death within a week from the same community
7. Two or more than two person of 2 years of age or older having watery stool more than 3 times in 24 h with sunken eye, fatigue or thirst or one death within a week
8. One or more cases of severe illness within 14 days of following vaccination or one death
9. One event of a natural or manmade disaster including earthquake, flood, landslide, avalanches, chemical, radiological, nuclear events, and active wars which can cause mass casualties
10. Sudden and unusual increase in disease or death of animals or poultries
11. Two or more severe cases and/or deaths with a similar type of symptoms within a week from the same community
Health facility
12. Severe illness among health care workers after caring for patients with similar symptoms
13. Two or more severe cases with the same symptoms within seven days and from the same location (e.g., household, camps, community unit, schools, factory, etc.)
14. A large, unexpected, sudden increase in admissions or deaths for any illness of the same type, including patients in intensive care units
15. Any suspected case of immediate notifiable disease
16. Any severe unexplained illness, including the unusual clinical presentation or a failure to respond to standard treatment
Veterinary services
17. Severe illness in veterinarians or community members after contact (culling, feeding, treating, vaccinating) with a sick or dead animal
18. A sudden increase in abortions among animals
19. All immediately notifiable diseases (brucellosis, anthrax, rabies, and influenza)
Laboratories
20. A pathogen that has not been detected for a long time in the country or a new pathogen
21. Large/sudden unexpected increase in numbers of specimens with the same testing request, or positive result for the same pathogen (including the pathogens that are multi-drug resistant including Tuberculosis)
22. Any pathogen on the immediately notifiable list
23. Un-subtypeable or new influenza or COVID-19 strain from a patient with Severe Acute Respiratory Infection (SARI)

### EBS flow of information

**Fig. 2:**
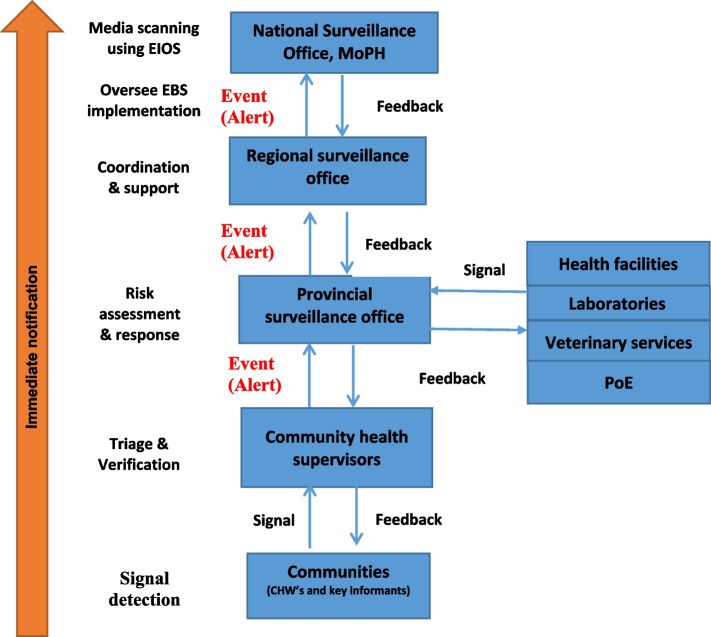
EBS flow of information in Afghanistan

The flow of information for the EBS (Fig. 2) was defined based on the steps of EBS and the current structure of the surveillance system. The flow of information relies mainly on key surveillance officers functioning at the local, provincial, regional and national levels as well as focal points from other relevant sources (laboratories, health facilities, veterinary services and PoEs).

In Afghanistan, the community signals should be notified immediately by CHWs and other key informants to the community health supervisors (CHSs). Meanwhile, signals from health facilities, veterinary services, laboratories, and PoEs should be reported immediately to the surveillance focal points at the provincial level. Each community signal will be triaged and verified within 24 h of detection by CHSs and by surveillance officers at the provincial level for health facility, laboratory, PoE and veterinary-related signals.

Triage involves evaluating two questions: (1) Is the reported information pertinent to early warning (i.e., could this signal indicate a genuine public health event)? and (2) Has the signal been previously reported (i.e., is it a duplicate)? Verification is conducted by addressing three questions: (1) Is the report accurate (i.e., is it truel)? (2) Has the information been provided by a trustworthy source or sources? and (3) Does the report fulfill the criteria for one or more of the already developed list of signals?. Once a signal is verified as true, reported by credible source and meet criterial for the list of signals, it is called event (alert).

The community-verified events must be immediately reported to the provincial surveillance officers to undergo a risk assessment and response process jointly with its RRTs. Meanwhile, provincial surveillance officers must triage and verify signals from the health facility, veterinary services, laboratories, and PoEs and if verified, should initiate the risk assessment and provide a response immediately in coordination with the provincial RRTs. High-risk events as indicated by the risk assessment process must be assessed or responded to within 48 h of notification.

To conduct risk assessment, the team need to formulate risk assessment questions. These questions will differ from one event to another. Examples of these questions are:

- Does the suspected disease have a high potential for spread?
- Is there higher than expected mortality and morbidity from the diseases?
- Is there a cluster of cases or deaths?
- Does the disease have possible consequences on trade or travel?
- Does the event affect livestock/wildlife?
- Are there any environmental consequences?

Once sufficient information has been collected, the risk assessment team must determine the the leve of risk and type of action. The WHO risk matrix should should be used for this purpose (Table 3).

**Table 3.**
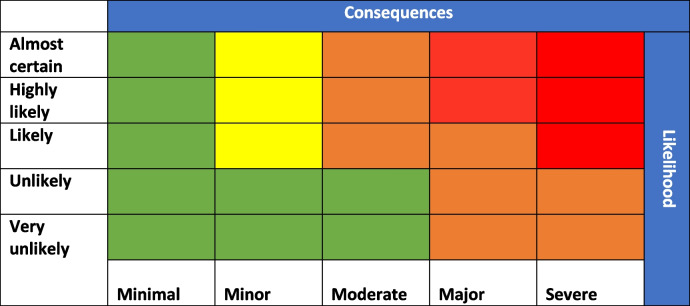
WHO Risk Matrix

Based on the level of risk the team should determine the outcome of risk assessment and actions needed. Table 4 describes the potential outcomes of the risk assessment based on information collected during the risk assessment process and assigned level of risk.

**Table 4:** Level of risk and possible outcomes with actions

Risk level	Action	Description	Criteria
**Low**	**No action needed**	Managed according to standard response protocols, routine control programs, and regulations.	The event does not constitute a risk to public health.
**Moderate**	**Monitor**	Roles and responsibility for the response must be specified	Serious consequences potentially exist.
			No human cases have been reported recently.
			Mortality/morbidity is as high as expected for the disease.
			Case severity is low.
**High**	**Immediate response**	Senior management attention is needed. There may be a need to establish command and control structures.	Mortality/morbidity is higher than expected.
			Diseases are unexpected/new to the community or health facility.
			There are possible consequences for trade and/or travel.
**Very high**		Immediate response is required even if the event is reported out of normal working hours. Immediate senior management attention is needed.	Case severity is high or higher than expected for the disease.
			Public health events cause significant panic in the community.
			The number of cases is increasing.

Feedback mechanism should be maintained through all levels, ensuring that all levels are aware of the actions has been take on the reported signal and events. This will not only essure that the system runs smoothly but also to enable trust and motivation at each levels.

The provincial surveillance officers should report EBS activities to the national team. The regional surveillance office is responsible for coordinating the EBS implementation in their provinces, providing training, conducting supervisory visits, and providing other operational support.

The central team of NDSR will lead the EBS implementation by coordinating the training of regional and provincial EBS personnel, supporting cascade training, providing training materials and SoPs, monitoring and evaluating EBS implementation, and conducting regular meetings to review data collected and provide feedback. The national surveillance office and the regional surveillance office are also responsible for giving support to the provincial-level surveillance officers in providing an appropriate response to the verified events in coordination with all other sectors.

Furthermore, the National Surveillance Officer is responsible for conducting regular monitoring of the media using the Epidemic Intelligence from Open Sources (EIOS) system, focusing on the prioritized list of events and diseases. During the verification process of signals detected through EIOS, the regional and provincial surveillance officers will serve as contacts. Once a signal is verified, the national surveillance officer will perform a rapid risk assessment and initiate a response.

### Developing operational EBS guidelines

After defining EBS information sources, prioritizing events, defining signals, and outlining the EBS information flow, EBS TWG developed a comprehensive operational guideline. This guideline integrates these steps and provides direction to EBS focal points at each level for effective implementation.

EBS TWG tailored Afghanistan’s EBS guidelines uniquely to its specific context, seamlessly integrating EBS into the existing NDSR structure and optimizing available human resources. They prioritized events based on the Afghan context, and carefully developed signals. These guidelines received technical support from the US CDC and WHO. Various guidelines, including WHO’s guidelines on EWAR with a specific focus on EBS and the US CDC’s EBS framework, served as references [4, 6].

The guideline itself is structured into three main sections, further subdivided into subsections, as elaborated in Table 5.

**Table 5:** Main Sections and subsections of the EBS guidelines

1. Introduction	1.1 Background
	1.2 Event-Based Surveillance
	1.3 Afghanistan’s National Disease Surveillance and Response (NDSR)
	1.4 Information Sources for Event-Based Surveillance
2. Guidelines for EBS implementation	2.1 EBS implementation in Afghanistan
	2.2 EBS at communities
	2.3 EBS at health facilities
	2.4 EBS at Veterinary Services
	2.5 EBS at laboratories
	2.6 EBS using EIOS
	2.7 EBS flow of information
	2.8 EBS Data Management
	2.9 Roles and responsibilities
3. Coordination and monitoring	3.1 Coordination and monitoring of EBS implementation in Afghanistan
	3.2 Monitoring and supervision

### Contextualizing the EBS training materials

In order to facilitate the training of EBS focal points at different levels (Fig. 2), training manuals were developed using the CDC-developed EBS training manual as a reference [6]. A total of five facilitator and corresponding participant training manuals exist to train focal points for EBS in communities, health facilities, laboratories, veterinary services, and at the provincial surveillance office.

### EIOS deployment

Media is an important source of information for EBS [7]. Afghanistan integrated media scanning as one of the surveillance functions under EBS. The EIOS tool has been deployed to facilitate the media scanning function in the country.

WHO proposes the EIOS tool to its member states to strengthen and solidify the surveillance system’s early detection, verification, assessment, and communication functions by utilizing publicly available, open-access information.

**Fig. 3:**
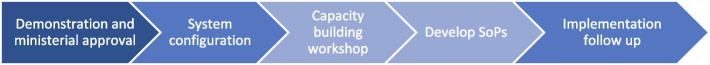
Steps of EIOS deployment in Afghanistan

After ministerial approval for the initiation of the EIOS, a rigorous configuration process was conducted to capture the local context and language. First, the priority diseases were identified, and for Afghanistan, the list was previously identified through the NDSR. Afterwards, the keywords were translated to the local languages, Pashto and Dari. The local news sources, social media (SM) accounts, and relevant websites were identified to be added to the sources list on the portal. Finally, a physical workshop was conducted to build the MoPH capacities to use EIOS. The steps of EIOS deployment are mentioned in Fig. 3.

Following the training, a close follow-up was conducted. The SoP for EIOS was developed and incorporated into the EBS guidelines.

### Pilot implementation

Following the development of country-specific guidelines, implementation commenced with a pilot phase in 7 provinces of the country by the end of 2022. The pilot focused on communities and health facilities. Implementation included training trainers for the 45 NDSR officers and coordinators at the national level, regional and provincial levels, with technical support from the WHO.

Subsequently, these trainers conducted training sessions for 883 medical doctors and CHSs in the piloted provinces. Following this, through third-party contractors, these medical doctors and CHSs further trained approximately 11,000 key informants at the community and health facility levels.

The pilot phase in the seven provinces yielded a total of 2,335 signals, of which 1,197 were verified as events. Risk assessments were conducted on 1,153 of these events.

## Discussion

The EBS in Afghanistan has advanced through several steps aimed at enhancing the country’s surveillance capabilities. This thorough process from assessment to selection of pilot sites, lasting about seven months, included extensive consultations at different levels. Key stakeholders under EBS CC and TWG, the US CDC, and the WHO provided valuable insights and expertise during the assessment and guideline development phases.

The approach to enhancing EBS in Afghanistan included several components that led to increased system efficiency and a more inclusive, structured system, despite the challenges posed by the country’s ongoing emergency situation. Afghanistan’s approach to EBS implementation can serve as a model for other countries facing similar contexts and aiming to establish EBS. One of the key strategies involved creating a roadmap for EBS and implementing it based on the results of a surveillance system landscape assessment. This approach ensured the integration of EBS into the existing structure of the surveillance system, which is the key for this work. The second strategy involved selecting key mentors from the national surveillance team and training them on EBS concepts from the very begining. This approach was crucial to ensure that MoPH took ownership of the system and led EBS efforts in the country.

Additionally establishing an EBS CC and a TWG fostered collaboration among diverse stakeholder groups and sectors. This collaboration aimed to collectively enhance the EBS system and broaden the sources of information for EBS in the country. The success of this approach can be seen in Vietnam [8], where the establishment of an EBS TWG resulted in improved alignment and reduced redundancies, as recommended in other published guidelines. The collaboration with other sectors for EBS also created political willness to implement the system which is key to highlight.

Finally, the development of SoP’s and signals for each identified information source under EBS establishes formalized reporting linkages among various important entities. One crucial linkage is between the human and animal health sectors, enhancing the detection of zoonotic events and advancing One Health efforts in the country [9–11]. Another significant linkage is the connection between communities and the health sector. Involving communities improves the detection of outbreaks, COVID-19 clusters and similar risks at the local level, leading to controlled transmission and reduced burden. Numerous countries have demonstrated improved risk detection by implementing community-based EBS [8, 12–14]. However, ensuring sustainability is essential when implementing EBS through communities. In Afghanistan, CHWs are integral to the community-based healthcare component of the Basic Package of Health Services (BPHS), which has been implemented for 20 years. NGOs train and supervise CHWs under government or United Nations Children’s Fund (UNICEF) contracts. The MoPH NDSR department coordinates with NGOs through the Community-Based Health Care (CBHC) department to support the surveillance system. CHWs, who are volunteers without incentives, work in pairs at health posts, offering services ranging from health promotion to referrals. They receive training from contracted NGOs for their surveillance roles. They received trainings for EBS through similar NGO’s.

In addition to communities, created linkages with health facilities and laboratories for EBS enables the detection of new emerging diseases and pathogens. EBS through these sources also enable detection of clusters of cases among health workers, which was challenging during the COVID-19 pandemic and resulted in increased transmission among health care workers. A systematic review targeted low and middle income countries similary highlights impotance of health facilities to strengthen the early warning function of national surveillance systems [15].

Importantly, this is the first time the country used the media as a source for EBS by deploying the EIOS platform. EIOS plays an important role in the detection of risks signaled through media [16]. EIOS also allows monitoring the health risks in bordering countries through published sources and having the control measures in place in response to the increased cross-border activities.

Afghanistan launched the pilot phase of EBS in seven provinces: Kabul (one zone), Herat, Kandahar, Nangarhar, Badakhshan, Balkh, and Bamiyan. This initial phase is focusing on communities and health facilities. Countries can choose to start implementing EBS from a single source and then expand to other sources by time based on workforce, resources, and infrastructure availability to ensure effectiveness and sustainability [6]. Afghanistan plans to introduce other sources after evaluating the current pilot phase.

The pilot phase provides insight into operationalizing elements of the guideline and SoP, identifying what does not work well, and offering guidance to adjust the EBS strategy for full-scale implementation in the country. The national surveillance office ensures the efficient implementation of EBS in Afghanistan through regular monitoring and evaluation activities. The monitoring and evaluation framework used in Vietnam for the pilot phase is utilized and developed a supervision visit checklist used at different levels responsible for EBS in Afghanistan [17]. For the final evaluation of the pilot phase, Afghanistan will adopt and use the Africa CDC Monitoring and Evaluation framework [18].

EBS implementation in Afghanistan encountered limitations such as delays in launching the pilot phase after selection of pilot sites due to political changes and the assignment of new EBS focal points. The Ministry collaborated closely with WHO to revitalize the EBS through training and consultations with the new mentors. With the changes since 2021, the use of EIOS has been limited, requiring training and onboarding of new focal points to replace those previously responsible. The TWG supporting the EBS had been inactive for an extended period and needs restructuring to support ongoing implementation and evaluation of the system for full countrywide implementation.

### Supplementary Information

**Supplementary Material 1.**

## Data Availability

No datasets were generated or analysed during the current study.
