# Supplementary material for: Strengthening event-based surveillance (EBS): a case study from Afghanistan
Source: Confl Health. 2024 Apr 30;18:39. doi: 10.1186/s13031-024-00598-1 (PMC11059675; doi:10.1186/s13031-024-00598-1)
Supplement: Supplementary file 1 — Supplementary Material 1. [file 13031_2024_598_MOESM1_ESM.docx]

**Supplementary 1. Landscape assessment Questionnaire**

**EMR Country Landscape Assessment: Integrated Disease Surveillance with a Focus on Event-Based Surveillance**

**GENERAL INFORMATION**

Date of Survey Completion: **_________________________**

| **Country** | **Country Rep Name** | **Title/Position** | **Organization** | **Email Address** |
| --- | --- | --- | --- | --- |
|  |  |  |  |  |
|  |  |  |  |  |
|  |  |  |  |  |

| **Facilitator Name** | **Title/Position** | **Organization** | **Email Address** |
| --- | --- | --- | --- |
|  |  |  |  |
|  |  |  |  |

**What is the purpose of this questionnaire?**

The objective of this questionnaire is to achieve a basic understanding of current surveillance structures, including those for COVID-19 surveillance with particular focus on event-based surveillance (EBS), of countries in the Eastern Mediterranean Region. Findings obtained from this questionnaire will aid in the design of tailored integrated surveillance platforms in country, including EBS.

**Who should participate in this questionnaire?**

Ministry of Health surveillance staff, including but not limited to event-based surveillance focal points, surveillance officers and other public health analysts, and Emergency Operations Center staff, should participate in this questionnaire. In some cases, WHO Country Office representatives may be asked to participate.

**How will this questionnaire be completed?**

CDC and WHO facilitators will schedule a 45-60-minute Zoom session with relevant country surveillance representatives to administer the questionnaire and discuss and record responses. Country representatives will be provided this questionnaire prior to the meeting in order to familiarize themselves with the nature of the questions. Completed questionnaires will not be shared outside of CDC, WHO, and the participating Ministry of Health.

**DEFINITIONS**

**Community-based surveillance (CBS):** The systematic detection and reporting of events of public health significance within a community by community members

**Early Warning and Response:** Early Warning and Response (EWAR) is defined as the organized mechanism to detect as early as possible any abnormal occurrence or any divergence from the usual or normally observed frequency of phenomena

**Event:** The International Health Regulations (IHR) define an event as *“[…] a manifestation of disease or an occurrence that creates a potential for disease; […]*” (which can include events that are infectious, zoonotic, food safety, chemical, radiological or nuclear in origin and whether transmitted by persons, vectors, animals, goods/food, or through the environment.).

**Event-based surveillance (EBS):** Defined by the World Health Organization (WHO) as the organized collection, monitoring, assessment and interpretation of mainly unstructured ad hoc information regarding health events or risks, which may represent an acute risk to human health.

**Healthcare facility event-based surveillance (HEBS):** Event-based surveillance that is conducted in hospitals and healthcare facilities. Healthcare workers are involved as primary reporting sources, such as during patient consultations, or as secondary sources reporting unusual health events or health risks picked up through patient consultations.

**Indicator-based surveillance (IBS):** Defined by WHO as the systematic (regular) collection, monitoring, analysis, and interpretation of structured data, i.e., of indicators produced by a number of well-identified, mostly health-based, formal sources.

**Internet event-based surveillance (IEBS):** Event-based surveillance that involves the systematic monitoring of information available in local and national media sources, including the Internet and social networks.

**Outbreak:** A disease outbreak is the occurrence of cases of disease in excess of what would normally be expected in a defined community, geographical area or season. An outbreak may occur in a restricted geographical area or may extend over several countries. It may last for a few days or weeks, or for several years. A single case of a communicable disease long absent from a population, or caused by an agent (e.g. bacterium or virus) not previously recognized in that community or area, or the emergence of a previously unknown disease, may also constitute an outbreak and should be reported and investigated.

**Response:** Any public health action triggered by the detection of a public health risk (e.g. monitoring of the *event*, information of the public, triggering field investigation and/or implementation of any control or mitigation measures). The nature of the response will have to be adapted according to the nature of the public health risk.

**Risk:** The likelihood of an event resulting in negative consequences for public health.

**Risk assessment:** A systematic process for gathering, assessing and documenting information to assign a level of risk to human health to an event. Risk assessment is conducted as part of an investigation of an event.

**Signals:** According to WHO, signals are data and/or information considered by the Early Warning and Response system as representing an event with potential acute risk to human health, such as an outbreak. Signals may consist of reports of cases or deaths (individual or aggregated), potential exposure of human beings to biological, chemical or radiological and nuclear hazards, or occurrence of natural or man-made disasters.

**Sub-national jurisdiction:** Sub-national jurisdictions may be defined differently in different countries. For the purpose of this questionnaire, a sub-national jurisdiction is the public health administrative level directly below the national-level, and may otherwise be referred to as regions, provinces, among others.

**Verification**: In the context of the IHR (article 1): *“[…] the provision of information by a State Party to WHO confirming the status of an event within the territory or territories of that State Party”.*(1) Under the IHR, all States Parties are required to provide verification upon request by WHO within a limited time period. In the current document, verification is also the pro-active crosschecking of the validity (veracity) of the signals collected by EWAR, by contacting the original source, additional sources, or by performing field investigation Verification requires that hoaxes, false rumors, and artefacts are eliminated from further consideration.

*Implementation of EWAR/EBS. Interim version: World Health Organization; 2014 (https://apps.who.int/iris/bitstream/handle/10665/112667/WHO_HSE_GCR_LYO_2014.4_eng.pdf;jsessionid=E99AE16819D5D4D4986464A7E2042CE2?sequence=1)*

*Protocol for Assessing National Surveillance and Response Capacities for the IHR (2005). Geneva: World Health Organization; 2010 (WHO/HSE/IHR/2010.7; http://www.who.int/ihr/publications/who_hse_ihr_201007_en.pdf?ua=1, accessed 31 March 2014).*

*International Health Regulations (2005), 2nd ed. Geneva: World Health Organization; 2008 (http://whqlibdoc.who.int/publications/2008/9789241580410_eng.pdf, accessed 31 March 2014).*

**QUESTIONNAIRE**

| **Detection** | | |
| --- | --- | --- |
| **Question #** | **Question** | **Response** |
| **1.** | A. Do you have a list of priority diseases, syndromes, and conditions under current national surveillance?  B. If yes, does it include COVID-19? | 🞏 Yes 🞏 No 🞏 Don’t know  🞏 Yes 🞏 No 🞏 Don’t know  Comments: |
| **2.** | Are standard case definitions available for all priority diseases, syndromes, and conditions under national surveillance? | 🞏 Yes 🞏 No 🞏 Don’t know  Comments: |
| **3.** | A. Which of the following sources contribute to routine case detection?  🞏 Public hospitals  🞏 Major/minor health centers  🞏 Local/village health services  🞏 Private sector  🞏Communities  🞏Community health workers/volunteers  🞏 Laboratories  🞏 NGOs  🞏 Others:  B. If yes for others, specify which ones: | 🞏Yes 🞏No 🞏Don’t Know  🞏Yes 🞏No 🞏Don’t Know  🞏Yes 🞏No 🞏Don’t Know  🞏Yes 🞏No 🞏Don’t Know  🞏Yes 🞏No 🞏Don’t Know  🞏Yes 🞏No 🞏Don’t Know  🞏Yes 🞏No 🞏Don’t Know  🞏Yes 🞏No 🞏Don’t Know  🞏Yes 🞏No 🞏Don’t Know  Comments: |
| **4.** | Is there a community-based surveillance (CBS) system in place to report COVID-19 cases, clusters, and deaths, or other unusual, unexpected or new events (e.g. high absenteeism at school, mass poultry die off, etc.)? | 🞏 Yes 🞏 No 🞏 Don’t know  Comments: |
| **5.** | If yes, explain the mechanism to capture the information (how information is collected, who is involved, at which level etc.) | Select format:  🞏 Paper 🞏 SMS/Phone  🞏 Computer/Internet 🞏 In person  Comments: |
| **6.** | Is there a health facility event-based surveillance (HEBS) system in place to report clusters of COVID-19 cases, illness in healthcare workers after treating COVID-19 cases, or other unusual, unexpected or new events? | 🞏 Yes 🞏 No 🞏 Don’t know  Comments: |
| **7.** | If yes, explain the mechanism to capture the information (how information is collected, who is involved, at which level etc.) | Select format:  🞏 Paper 🞏 SMS/Phone  🞏 Computer/Internet 🞏 In person  Comments: |
| **8.** | Is there an internet event-based surveillance system (IEBS) in place to scan for unusual, unexpected or new health events from media sources? | 🞏 Yes 🞏 No 🞏 Don’t know |
| **9.** | If an IEBS system is in place, is there a checklist of sources to guide internet scanning? | 🞏 Yes 🞏 No 🞏 Don’t know |
| **10.** | If an IEBS system is in place, is there a media scanning system/software available to automate IEBS workflow for human analysts? | 🞏 Yes 🞏 No 🞏 Don’t know |
| **11.** | Do the following sources contribute to routine signal detection?  🞏 Printed or electronic media reports  🞏 Radio broadcasts  🞏 Social media platforms  🞏 Community health workers (or equivalent)  🞏 NGOs  🞏 A hotline or call center  🞏 WhatsApp or SMS messaging  🞏 Others, specify which ones: | 🞏Yes 🞏No 🞏Don’t Know  🞏Yes 🞏No 🞏Don’t Know  🞏Yes 🞏No 🞏Don’t Know  🞏Yes 🞏No 🞏Don’t Know  🞏Yes 🞏No 🞏Don’t Know  🞏Yes 🞏No 🞏Don’t Know  🞏Yes 🞏No 🞏Don’t Know  Comments: |
| **12.** | A. Are there regular team meetings to discuss new signals detected through the IEBS system and appropriate follow ups?    B. If yes, please describe their frequency. | 🞏 Yes 🞏 No 🞏 Don’t know  Comments: |
| **13.** | If any EBS system is currently in place (i.e. CBS, HEBS and/or IEBS), have SOPs or guidelines been established? | 🞏 Guidelines 🞏 SOPs 🞏 Neither  🞏 Don’t know  Comments: |
| **14.** | Do you receive weekly surveillance reports from the sub-national jurisdiction? | 🞏 Yes 🞏 No 🞏 Don’t know  Comments: |
| **15.** | Do the sub-national jurisdictions use a standardized form to send weekly surveillance reports? | 🞏 Yes 🞏 No 🞏 Don’t know  Comments: |
| **16.** | How does the sub-national jurisdiction report to you weekly?  🞏 Send a hard copy of the form  🞏 Send a copy of the form by internet  🞏 Phone call  🞏 SMS/WhatsApp  🞏 Enter data into an electronic tool, specify which tool:  🞏 Other, please specify: | Comments: |
| **17.** | Does your organization communicate with the animal sector (e.g. farms, veterinary services, etc.) to exchange surveillance information? | 🞏 Yes 🞏 No 🞏 Don’t know |
| **18.** | What is the principal method used for COVID-19 detection in your country? Please describe briefly, including surveillance systems used: | Comments: |
| **Registration** | | |
| **19.** | A. Do you have a signal log?  B. If yes, do all sub-national levels use the signal log? | 🞏 Yes 🞏 No 🞏 Don’t know  🞏 Yes 🞏 No 🞏 Don’t know |
| **20.** | If a signal log is available, specify which type of information is registered:  Check all that apply:  🞏 Date/time of detection  🞏 Date/time of first case reported  🞏 Initial agent/syndrome reported  🞏 Clinical description of illness  🞏 Number of cases/deaths reported  🞏 Location of report (specify granularity of admin level)  🞏 Source of report  🞏 Others? List here: | Comments: |
| **21.** | Is there a database with routine aggregated data sent from the sub-national jurisdictions at your level? | 🞏 Yes 🞏 No 🞏 Don’t know |
| **22.** | A. Do you have a centralized electronic event management system (EMS) to keep track of event reporting from all administrative levels?  B. If yes, does it include the following stages:  🞏 Date of first reported case (outbreak start)  🞏 Date of signal detection by EBS  🞏 Date of outbreak notification (first reported to public health authorities)  🞏 Date of event verification  🞏 Date of laboratory confirmation  🞏 Date of public health intervention  🞏 Date of first official release of information to the public  🞏 Date of event updates  🞏 Date of end of outbreak  🞏 Others? List here: | 🞏 Yes 🞏 No 🞏 Don’t know |
| **23.** | If there is a centralized electronic event management system (EMS), is it interoperable with the national surveillance system architecture (e.g. DHIS-2, SORMAS, other?)? Please specify. | 🞏 Yes 🞏 No 🞏 Don’t know  Comments: |
| **24.** | If there is a centralized electronic event management system (EMS), who manages the inputs into the system?  Who is responsible for the prospective tracking of events entered into the system? | Comments: |
| **Signal Verification** | | |
| **25.** | What is the timeframe used within which signals should be verified after detection? | Comments: |
| **26.** | Do you maintain a list of subject matter experts (SMEs)/networks that specialize in particular disease areas who might be called to assist with the signal verification? | 🞏Yes, for all  🞏Yes, for some  🞏 No  🞏 Don’t know |
| **27.** | Is there national laboratory capacity to meet diagnostic and confirmatory laboratory requirements for priority diseases? | 🞏 Yes 🞏 No 🞏 Don’t know  Comments: |
| **Data Analysis and Interpretation** | | |
| **28.** | Are data collected through routine surveillance analyzed on a regular basis to detect the deviations from the alert/epidemic thresholds? | 🞏 Yes 🞏 No 🞏 Don’t know |
| **29.** | Do predefined action thresholds for selected diseases exist? | 🞏Yes, for all diseases  🞏Yes, for some diseases  🞏 No  🞏 Don’t know |
| **30.** | What is utilized for analyzing and/or interpreting surveillance data (e.g. Epi Info, Excel, SAS, STATA, R, Tableau)? | Comments: |
| **Risk Assessment** | | |
| **31.** | Do you perform a risk assessment following detection of every new active public health event? | 🞏 Yes 🞏 No 🞏 Don’t know  Comments: |
| **32.** | A. Do you use a specific tool for risk assessment?  B. If yes, which tool? | 🞏 Yes 🞏 No 🞏 Don’t know  Comments: |
| **33.** | Is Annex II of the IHR referenced to determine if an event under risk assessment is notifiable to the WHO? | 🞏 Yes 🞏 No 🞏 Don’t know |
| **Reporting** | | |
| **34.** | If EBS is occurring, are routine EBS reports disseminated from the central level? | 🞏 Yes 🞏 No 🞏 Don’t know |
| **35.** | If yes, please provide details as to whom is receiving these reports. | Comments: |
| **36.** | If yes, please provide details as to the frequency of reporting. | Comments: |
| **37.** | If yes, please describe how these reports are disseminated. | Comments: |
| **38.** | Is there a national IHR focal point? | 🞏 Yes 🞏 No 🞏 Don’t know |
| **39.** | Are there regional or bilateral arrangements with neighboring countries to share/report surveillance or events data? | 🞏 Yes 🞏 No 🞏 Don’t know |
| **Epidemic Preparedness** | | |
| **40.** | Is there a national public health emergency response plan at the central level? | 🞏 Yes 🞏 No 🞏 Don’t know  Comments: |
| **Response and Control** | | |
| **41.** | Is there a public health emergency operations center or intelligence hub at the national level? | 🞏 Yes 🞏 No 🞏 Don’t know  Comments: |
| **42.** | Are incident management systems established for management, communication, and control during public health emergency response operations at the central level? | 🞏 Yes 🞏 No 🞏 Don’t know  Comments: |
| **43.** | Are there multidisciplinary Rapid Response Teams (RRTs) at your level to respond to acute public health events? | 🞏 Yes 🞏 No 🞏 Don’t know  Comments: |
| **Communication Tools** | | |
| **44.** | Does the facility have trouble maintaining reliable internet access? | 🞏 Yes 🞏 No🞏 Don’t know |
| **45.** | If yes, how often is internet access disrupted? (Hourly, daily, weekly, monthly?) | Comments: |
| **46.** | Is access to a phone network available 24/7?  If no, describe access: | 🞏 Yes 🞏 No🞏 Don’t know  Comments: |
| **47.** | Is electricity available 24/7? | 🞏 Yes 🞏 No🞏 Don’t know |

**Supplementary 2. Supervision checklist developed for monitoring and evaluation of EBS**

## Annex 7: Recommendations for conducting supportive supervision

**Purposes:**

1. To determine if EBS is operating as intended in the implementation guide
2. The success and challenges of implementing EBS
3. Improve the quality of the EBS activities

**Actors**

| **Supervisor** | **Supervisee** |
| --- | --- |
| National surveillance office | Regional, provincial, and health facilities |
| Regional surveillance officer | Provincial surveillance officers and health facilities |
| Provincial surveillance officers | Health facilities, laboratories, and communities |
| Surveillance focal points at sentinel sites | Communities, key informants, and CHW’s |

**Frequency of the supervision**

Supportive supervision visits must be quarterly by the national and regional level, and monthly by the provincial level.

Need-based monitoring visits can be done when required.

**Methods**

Before visit

- Develop plans and timelines for supportive supervision visits, and assign responsibilities to supervisors
- Prepare the supervision checklist and other related documents
- Review the results of previous supervisory visits

At the visit

- Use the supervision checklist (Annex 8) to interview the EBS focal points
- Review the signal and health facilities registers
- Discuss outstanding issues, difficulties, and challenges during the implementation of the EBS and propose solutions to address them
- Provide on the job training and orientation

After the visit

- Produce a summary report and provide feedback to the supervisees
- Ensure follow-up and assist in solving problems discovered during the site visit.
- Archive supervision checklist for future formative supervision visits

## Annex 8: EBS Supervision Checklist

| **EBS Supervision Checklist** | |
| --- | --- |
| **Province/District:** | **Name of supervisor:** |
| **Name of facility:** | **Name of supervisee (EBS responsible):** |
| **Date of supervision:** | **Date of last supervision:** |

| # | Questions to be asked during each supervision visit: | Source of information | Answers | | | Why not? | Recommended actions |
| --- | --- | --- | --- | --- | --- | --- | --- |
|  |  |  | Yes | No | NA |  |  |
| 1 | Has the health facility designated someone as the focal point person for EBS?  *If yes, Name:* __________________________________  *(The focal point for EBS should be the person answering these questions)* | Surveillance focal points at sentinel sites and EBS focal points at non-sentinel sites |  |  |  |  |  |
| 2 | Has the staff responsible for EBS changed since the last supportive supervision visit?  *If yes, name of new EBS staff:* |  |  |  |  |  |  |
|  | Has new staff received EBS training? |  |  |  |  |  |  |
|  | When was the most recent date when they received EBS training? _________________ |  |  |  |  |  |  |
| 3 | Does the focal point know the list of health facility signals that are required to be reported? |  |  |  |  |  |  |
| 4 | Does the healthcare facility have a poster of health facility signals that are required to be reported? |  |  |  |  |  |  |
| 5 | Does the facility have the necessary equipment available to carry out its EBS reporting duties?   1. Phone 2. Internet 3. Computer |  |  |  |  |  |  |
| 6 | Can you provide the name and contact information (phone, email) of the provincial surveillance officer that signals can be reported?  Name & Phone: __________________________ |  |  |  |  |  |  |
| 7 | How many signals have been reported since the last month? |  |  |  |  |  |  |
|  | Review the EBS Logbook for the last month:  How many signals/events were recorded in the logbook for the last month? |  |  |  |  |  |  |
|  | Is the signal information recorded in the logbook complete for all signals? |  |  |  |  |  |  |
|  | If no, please describe which information is missing: __________________________ |  |  |  |  |  |  |
| 8 | Were any training activities related to event-based Surveillance provided to facility staff during the last month? |  |  |  |  |  |  |
| 9 | Were any challenges faced in detecting signals? If yes, describe these challenges: |  |  |  |  |  |  |
|  | Were any challenges faced in reporting of signals/events? If yes, describe these challenges: |  |  |  |  |  |  |
| 10 | Other comments and notes for follow-up and/or to check on during next supervisory monitoring visit: ______________________ |  |  |  |  |  |  |
| 11 | Are there plans for educating the community about recognizing and reporting the community-based signals? | Surveillance focal points at sentinel sites |  |  |  |  |  |
| 12 | Were any training activities related to event-based surveillance provided to the community in this province during the last month? |  |  |  |  |  |  |
| 13 | Do you have the necessary equipment available to carry out the triage, verification, and reporting of the CBS signals in addition to the HF signals?   1. EBS guideline 2. List of signals 3. Triage and verification tool |  |  |  |  |  |  |
| 14 | How many signals have been reported by the community since the last visit? |  |  |  |  |  |  |
|  | How many of these signals were verified as events? |  |  |  |  |  |  |
|  | Were the verified events immediately reported to the intermediate level for risk assessment and response? |  |  |  |  |  |  |
|  | Was there any challenge in reporting these events to the provincial surveillance office? |  |  |  |  |  |  |
| 15 | How are the triaged and verified signals are recorded in your facility? |  |  |  |  |  |  |
|  | Is the recorded information complete? If no which information is missing? |  |  |  |  |  |  |
| 16 | Were any supervision visits related to CBS conducted at the community level during the last month?  If yes, please explain the detail of the visit and outcome |  |  |  |  |  |  |
| 17 | Do the focal points have the necessary equipment available to carry out their EBS duties?   1. EBS Guideline 2. Triage, verification, risk assessment, and response tools 3. Access to DHIS2 and guidance to record the EBS data | Provincial surveillance focal points |  |  |  |  |  |
| 18 | In the past month, how many events have benefited from a risk assessment? |  |  |  |  |  |  |
|  | How many risk assessments were completed within 48 hours? |  |  |  |  |  |  |
|  | Was an immediate response provided to high-risk events? |  |  |  |  |  |  |
| 19 | Was there any challenge in responding? |  |  |  |  |  |  |
| 20 | Are all risk assessment reports available? |  |  |  |  |  |  |
|  | Are the reports well archived? If so check |  |  |  |  |  |  |
|  | Is the EBS database (DHIS2) properly informed?  Check the completeness and quality of the data. |  |  |  |  |  |  |
| 21 | Have you provided feedback on the signals and events reported to you in the previous month? If so check |  |  |  |  |  |  |
| 22 | Did you or the surveillance office visit the facilities/laboratories implementing EBS in the last month? If yes, record the key finding and review the completed checklists and/or reports:  What actions (if any) were taken? ……………………………………………………… |  |  |  |  |  |  |
| 23 | Is there a schedule of the dates for monitoring visits to each Health Facility/Laboratory? |  |  |  |  |  |  |
| 24 | Do you know that you can report some patterns of disease or other information that put people’s health at risk to health authorities?  ----------------------------------------  How did you become aware of this?   1. Oriented by health authorities 2. Communicated by a friend, colleague, or religious leader 3. Posters of the list of signals been distributed   If been oriented by health authorities, when was the last time? ------------------- | Communities (Key informants and CHW’s) |  |  |  |  |  |
| 25 | When you identify a signal, how do you report the signal to the public health system?  Can you provide the contact information (phone) of the health authority or the address (if reporting in person) that signals can be reported?  Phone and location: ___________________ |  |  |  |  |  |  |
| 26 | Have you or other members reported any signal to the health authorities since last month?  Was the result of the signal been communicated to you by health authorities? Or any other relevant feedback? ………………….. |  |  |  |  |  |  |
| 26 | Do you have any problem reporting the signals to health authorities or any problem with detecting the signals in your community? ………………………. |  |  |  |  |  |  |
| 27 | Please check if the community you are visiting has a poster of signals around?  ………………………..  Note: Please take some posters with you for distribution |  |  |  |  |  |  |
| 28 | Do you exchange the information on the list of signals, detection, and reporting to the other community members? -------------------------------------------------  **At the end of the visit:**   1. Discuss outstanding issues, difficulties, and challenges for detection and reporting of signals by community members and propose solutions to address them 2. Provide orientation on the list of signals, detection, and reporting 3. Encourage the interviewee to sensitize other community members on the list of community signals, detection, and reporting |  |  |  |  |  |  |

**Supplementary 3. List of notifiable disease for IBS from sentinel sites on a weekly basis**

1. **Acute Respiratory Tract Infection (ARI cough & cold), No Pneumonia – including ILI:**

Acute onset of cough, cold, coryza (runny nose), pharyngitis, laryngitis, bronchitis, or bronchiolitis with or without fever.

**Influenza-Like Illness (ILI)**:

An acute respiratory infection with:

- measured fever of ≥ 38 C°;
- and cough;
- with onset within the last 10 days.

1. **ARI – Pneumonia including SARI:**
   1. In adults: ARI, fever and crepitation or bronchial sounds on chest auscultation.
   2. Severe Pneumonia in Children under 5, the clinical case definition (IMCI):

A child presenting with cough or difficult breathing and:

- 50 or more breaths per minute for infant age 2 months up to 1 year
- 40 or more breaths/minute for young child 1 up to 5 years.

Note: A young infant age 0 up to 2 months with cough and fast breathing is classified in IMCI as “serious bacterial infection” and is referred for further evaluation.

Clinical case definition (IMCI) for severe pneumonia:

A child presenting with cough or difficult breathing and any general danger sign, or chest indrawing or stridor in a calm child. General danger signs for children 2 months to 5 years are: unable to drink or breast feed, vomits everything, convulsions, lethargy, or unconsciousness.

**Confirmed case:** Radiographic or laboratory confirmation of pneumonia may not be feasible in most districts.

**Severe Acute Respiratory Infection (SARI)** (for all age groups)**:** An acute respiratory infection with history of fever or measured fever of ≥ 38 C°; and cough; with onset within the last 10 days; and requires hospitalization.

1. **COVID-19 Case Definitions:** (Suspected cases should be reported when there is widely community transmission of virus, otherwise only confirmed cases should be reported by the Health Facilities)

**Suspected COVID-19 Case:**

1. ***A person who meets the clinical AND epidemiological criteria:***

**Clinical Criteria:**

- Acute onset of fever AND cough; OR
- Acute onset of **ANY THREE OR MORE** of the following signs or symptoms:

Fever, cough, general weakness/fatigue, headache, myalgia, sore throat, coryza, dyspnoea, anorexia/nausea/vomiting, diarrhoea, altered mental status.

**AND**

**Epidemiological Criteria:**

- Residing or working in an **area with high risk of transmission of virus:** closed residential settings, humanitarian settings such as camp and camp-like settings for displaced persons; any time within the 14 days prior to symptom onset; or
- Residing or travel to an **area with community transmission** any time within the 14 days prior to symptom onset; or
- Working in **any health care setting**, including within health facilities or within the community; anytime within the 14 days prior of symptom onset.
  - 1. ***A patient with severe acute respiratory illness:***

(SARI: acute respiratory infection with history of fever or measured fever of ≥38C°; and cough; with onset within the last 10 days; and requires hospitalization).

- - 1. ***Asymptomatic person not meeting epidemiologic criteria with a positive SARS-CoV-2 Antigen-RDT***

**Probable COVID-19 Case:**

1. A patient who meets clinical criteria above AND is a contact of a probable or confirmed case, or linked to a COVID-19 cluster
2. A suspect case with chest imaging showing findings suggestive of COVID-19 disease
3. A person with recent onset of **anosmia** (loss of smell) or **ageusia** (loss of taste) in the absence of any other identified cause.
4. **Death**, not otherwise explained, in an adult with **respiratory distress** preceding death AND **was a contact of a probable or confirmed case** or linked to a **COVID-19 cluster**

**Confirmed COVID-19 Case:**

1. A person with a positive **Nucleic Acid Amplification Test (NAAT)**
2. A person with a **positive SARS-CoV-2 Antigen-RDT AND** meeting either the **probable case definition or suspect criteria A OR B**
3. An **asymptomatic person with a positive SARS-CoV-2Antigen-RDT** who is a **contact of a probable or confirmed** case
4. **Acute Watery Diarrhea (AWD) without dehydration:**

Acute watery diarrhoea is an illness characterized by 3 or more loose or watery (non-bloody) stools within 24-hour without dehydration.

1. **Acute Watery Diarrhea (AWD) with Dehydration:**

**Suspected Cholera:**

- 1. ***In areas where a cholera outbreak has not been declared:*** *Any patient aged 2 years*

and older presenting with acute watery diarrhoea and severe dehydration or dying from acute watery diarrhoea.

- 1. ***In areas where a cholera outbreak is declared*:** any person presenting with or dying from acute watery diarrhoea.

**Confirmed cholera case**: A suspected case with Vibrio cholerae O1 or O139 confirmed by culture or PCR.

1. **Acute Bloody Diarrhea (Shigellosis): (**Acute Diarrhea with visible blood in the stool (preferably observed by a clinician rather than solely from the patient’s report).
   1. **Suspected Shigellosis:** Bloody diarrhea, fever, stomach cramps in 5 or more connected cases.
   2. **Confirmed case:** Isolation of *Shigella dysenteriae*
2. **Suspected Meningitis:**

Any person having sudden onset of fever (>38°C axillary) and one or more of the following:

- Neck stiffness
- Altered consciousness
- Other meningeal sign or petechial or purpural rash
- In infants under one year of age, suspect meningitis when fever is accompanied by bulging fontanelle.

1. **Acute Jaundice Syndrome:**

Acute onset of jaundice (yellowing of whites of eyes or skin or dark urine) AND severe illness with or without fever AND the absence of any known precipitating factors

1. **Suspected Measles:**

Any person with fever **AND** maculopapular (non-vesicular) generalized rash **AND ONE** of the following: cough, runny nose (coryza) or red eyes (conjunctivitis)

**OR** any person in whom a clinician suspects measles

**Confirmed case:** Suspected case with positive serum IgM and no measles vaccination in prior 28 days.

1. **Suspected Pertussis:** A person with a cough lasting at least two weeks with one of the following: Paroxysms (i.e. fits) of coughing; or inspiratory “whoop”; or post-tussive vomiting (i.e. vomiting immediately after coughing) AND without other apparent cause.
2. **Probable/Suspected Diphtheria:** An acute illness characterized by an adherent membrane on the tonsils, pharynx and/ or nose and any one of the following: laryngitis, pharyngitis or tonsillitis.
3. **Neonatal Tetanus:**
   1. **Suspected case:** Any neonatal death between 3 and 28 days of age in which the cause of death is unknown or any neonate reported as having suffered from neonatal tetanus between 3 and 28 days of age and not investigated.
   2. **Confirmed case:** Any neonate with normal ability to suck and cry during the first 2 days of life, and who between 3 and 28 days of age cannot suck normally and becomes stiff or has convulsions or both. Hospital-reported cases are considered confirmed.
4. **Acute Flaccid Paralysis (AFP):** Sudden floppy paralysis or muscle weakness in a child aged < 15 years due to any cause, including Guillain Barré syndrome, or any person with paralytic illness at any age when polio is suspected by a clinician.
5. **Confirmed Malaria:**

Positive laboratory confirmation by blood smear or rapid diagnostic test for malaria

1. **Suspected Typhoid Fever:**

Any person with gradual onset of steadily increasing and then persistently high fever with or without additional clinical features such as chills, malaise, headache, sore throat, cough, abdominal pain, constipation, or diarrhoea.

Or

A physician’s suspicion of enteric fever (typhoid or paratyphoid)

**Confirmed case**:

Suspected case laboratory confirmed by culture or molecular methods of *Salmonella Typhi* from blood, bone marrow, bowel fluid or stool.

1. **Suspected Dengue Fever:**

Clinical case definition (suspected dengue fever):

Any person having acute onset of fever (> 380C) for 2-10 days with at least two of the following manifestations: severe headache, retro-orbital pain, myalgia/ arthralgia, positive tourniquet test.

**Probable:** A suspected case and one or more of the following:

• Supportive serology (reciprocal haemagglutination-inhibition antibody titre >=1280, comparable IgG EIA titre or positive IgM antibody test in late acute or convalescent-phase serum specimen).

• Occurrence at same location and time as other confirmed cases of dengue fever.

**Confirmed**: A suspected case, laboratory-confirmed, by one or more of the following:

• Isolation of the dengue virus from serum, plasma, leukocytes, or autopsy samples

• Demonstration of a fourfold or greater change in reciprocal IgG or IgM antibody titres to one or more dengue virus antigens in paired serum samples

• Demonstration of dengue virus antigen in autopsy tissue by immunohistochemistry or immunofluorescence or in serum samples by EIA

• Detection of viral genomic sequences in autopsy tissue, serum or CSF samples by polymerase chain reaction (PCR).

1. **Acute Hemorrhagic Fever Syndrome:** (Acute hemorrhagic fever syndromes can be attributable to dengue (dengue hemorrhagic fever), Ebola-Marburg viral diseases, Lassa fever, yellow fever, Rift Valley fever, hantavirus infections, Crimean-Congo hemorrhagic fever, and other viral, bacterial or rickettsial diseases with a potential to produce epidemics.

**Clinical case description:** Acute onset of fever of less than 3 weeks duration in a severely ill patient AND any TWO of the following signs:

- hemorrhagic or purpuric rash
- bleeding from the nose (epistaxis)
- vomiting blood (haematemesis)
- coughing up blood (haemoptysis)
- blood in stools
- other hemorrhagic symptom and absence of predisposing host factors for hemorrhagic manifestations
